# Aminated PET Thin Film as a Functionalized Insulating Layer for Capacitive Gliadin Aptasensor Construction

**DOI:** 10.3390/bios16060324

**Published:** 2026-06-03

**Authors:** Po-Chung Chen, Po-Chuan Hsieh

**Affiliations:** 1 Department of Biomechatronic Engineering, National Ilan University, Yilan City 260007, Taiwan; chenpc@niu.edu.tw; 2 Department of Biomechatronics Engineering, National Taiwan University, Taipei 106319, Taiwan

**Keywords:** aminolysis, label-free, reagentless, regeneration, gluten, soy sauce

## Abstract

Celiac patients require strict avoidance of gliadin, the primary immunotoxic component of gluten, making sensitive detection essential for food safety. A label-free and reagentless capacitive aptasensor for gliadin detection was developed using an aminated polyethylene terephthalate (PET) thin film as both an insulating layer and functionalization platform. The PET surface was modified via ethylenediamine-mediated aminolysis, enabling covalent immobilization of 5′-NH_2_-modified gliadin aptamers through glutaraldehyde crosslinking. Under optimized conditions (23 µm initial PET thickness and 10 µM aptamer), the sensor showed a linear response from 10 to 500 µg/mL gliadin (R^2^ = 0.9792), with a detection limit of 6.0 µg/mL, equivalent to 12 ppm gluten, which is well below the regulatory threshold of 20 ppm for gluten-free labeling. The aptasensor showed excellent correlation with commercial ELISA for 20 gluten-containing soy sauce samples (R^2^ = 0.926) and spike recoveries of 91.7–105.7% in two gluten-free products. Efficient regeneration was achieved with 25 mM arginine (pH 9.0), retaining >80% activity after six cycles. This simple, low-cost, and reusable platform relies solely on a single PET thin film as consumable in a custom-built system with lab-friendly aminolysis conditions. It substantially lowers barriers to functionalized insulating layer fabrication, the primary challenge in capacitive aptasensor development, providing a promising method for on-site gliadin monitoring in gluten-free food safety applications.

## 1. Introduction

Celiac disease affects approximately 1% of the global population and is driven by gliadin, a principal immunotoxic component of gluten that induces small intestinal damage upon ingestion. Therefore, strict compliance with a gluten-free diet is necessary, with gluten-free labeling defined by a regulatory threshold of 20 ppm gluten (equivalent to 10 ppm gliadin) [1,2,3,4]. However, recent studies indicate that gluten contamination continues to be detected in products labeled as gluten-free, with non-compliance rates of 11–36% exceeding 20 ppm globally (up to >100-fold in some cases), even in naturally gluten-free foods like maize, rice, and oats [5,6,7,8]. Despite regulatory advances, cross-contamination remains a critical food safety issue for celiac patients, underscoring the need for reliable, highly sensitive, simple analytical methods for routine use.

Conventional gliadin assays, such as ELISA, are highly accurate but remain laborious, time-consuming, and impractical for on-site applications. In contrast, aptasensors provide distinct advantages over antibody-based systems, including superior stability, lower cost, rapid response times, and robust performance in complex food matrices [9,10,11,12]. These benefits stem from the strong affinity of aptamers for protein targets, mediated by molecular shape complementarity, electrostatic interactions, hydrogen bonding, hydrophobic interactions, and van der Waals forces [13,14]. Electrochemical and colorimetric aptasensors have advanced substantially for gliadin detection, offering robust, antibody-independent alternatives for food safety monitoring. Amaya-González et al. selected unmodified DNA aptamers targeting the immunodominant 33-mer peptide of gliadin. These aptamers enabled a competitive electrochemical magnetoassay using streptavidin–HRP as the label and H_2_O_2_/TMB as the redox substrates, attaining a limit of detection (LOD) of 0.5 ng/mL for gliadin with negligible cross-reactivity toward nontoxic proteins [15]. López-López et al. developed a disposable competitive electrochemical aptasensor on a screen-printed carbon electrode. Using the same streptavidin–HRP label and H_2_O_2_/TMB enzymatic reaction, the sensor attained an LOD of 0.113 µg/mL gliadin and showed strong agreement with a commercial ELISA in real food samples [16]. Malvano et al. introduced a label-free impedimetric aptasensor based on a ferri/ferrocyanide redox couple, yielding an LOD of 5 µg/mL gliadin and good concordance with ELISA results in both gluten-containing and gluten-free products [17]. Ramalingam et al. further advanced this approach by integrating a MoS_2_/graphene/Au nanocomposite with a PDMS-based flexible microfluidic biochip fabricated on a screen-printed carbon electrode. Using a ferri/ferrocyanide redox indicator, the platform enabled electrochemical detection of gliadin with an LOD of 7 pM, a 20 min assay time, and good selectivity and stability in real flour samples [18]. Svigelj et al. rationally truncated a gliadin aptamer to minimize self-hybridization. This modification enabled a sandwich-format electrochemical sensor that operates directly in the deep eutectic solvent ethaline, again using streptavidin–HRP and H_2_O_2_/TMB. The sensor demonstrated sufficient sensitivity to detect 20 µg/kg gluten in food after simple extraction [19]. Svigelj et al. further refined this approach into a label-free impedimetric aptasensor on a gold nanoparticle (AuNP)-modified interface using the truncated aptamer and ferri/ferrocyanide as the redox probe. The platform achieved an LOD of 0.05 µg/mL for gliadin and successfully quantified the target in hydrolyzed matrices [20]. Colorimetric formats have also progressed toward instrument-free operation. Ham et al. developed a label-free AuNP–aptamer biosensor in which gliadin-induced aggregation in NaCl solution produces a visible color change (LOD = 32.1 ng/mL). The sensor performed reliably in complex matrices including pasta, bread, and cookies [21]. Qin et al. integrated a similar AuNP–aptamer colorimetric strategy with smartphone-based RGB analysis, achieving an LOD of 12 pM and spiked recoveries of 85–122% in grain alcohol extracts [22]. Despite meeting the selectivity and sensitivity requirements for gluten-free product monitoring, these designs still rely on enzyme labeling, the addition of electroactive redox reagents, or AuNPs as chromogenic agents. This dependence limits simplicity and cost-effectiveness, hindering their translation into point-of-care testing devices.

Capacitive biosensors enable fully label-free, reagentless detection using simple electronics to track target–biorecognition binding dynamics in real time, typically within tens of minutes. A major challenge in capacitive aptasensing is the development of a highly insulating yet chemically reactive layer that ensures stable baseline capacitance and efficient aptamer immobilization. Tsai et al. recently addressed this challenge by using a parylene double-layer coated screen-printed electrode. The coating provided robust insulation and surface functionalization, yielding a linear capacitance response to gliadin (10–1000 µg/mL; LOD = 7 µg/mL) over a 20 min monitoring period that closely matched commercial ELISA results [23]. This simple, low-cost, scalable, and rapid platform is ideal for routine, field-deployable verification of gluten-free products. However, the low aspect ratio of commercial screen-printed interdigitated electrodes imposes significant constraints. To maximize preservation of the interelectrode cavity for ultrasensitive detection, only an ultrathin parylene coating deposited via chemical vapor deposition (CVD) facilitates successful capacitive aptasensing, thereby precluding alternative insulating layer fabrication methods, owing to both technical limitations and cost-effectiveness concerns. Moreover, parylene CVD entails substantial drawbacks, necessitating operation at several hundred °C under high vacuum with sustained argon flow for several hours, which renders the process both energy-intensive and time-consuming.

To overcome the limitations of parylene coatings and electrode patterning described above, this study introduces a commercially available, inexpensive polyethylene terephthalate (PET) thin film as the insulating layer on a custom-built, acrylic block based parallel-plate capacitive sensing platform. The PET surface was aminated via ethylenediamine-mediated aminolysis, a proven method for generating NH_2_ groups on inert PET [24]. This enabled covalent immobilization of 5′-NH_2_-modified aptamers through glutaraldehyde crosslinking to fabricate a capacitive gliadin aptasensor. In this custom-built system, the only consumables required are a single PET thin film and two layers of double-sided tape, while the aminolysis reaction employs simple, lab-friendly conditions executable in any chemistry laboratory. These novel features substantially lower the barriers to fabricating functionalized insulating layers, the major challenge in capacitive aptasensor development. Here, key performance metrics were systematically evaluated, including PET film thickness, aptamer dosage, matrix tolerance in soy sauce (a common gluten-containing food product), accuracy versus commercial ELISA kits, and aptamer regeneration efficiency.

## 2. Materials and Methods

### 2.1. Reagents and Samples

Ethanol, glutaraldehyde, glycine, HCl, NaCl, MgCl_2_, NaH_2_PO_4_, Na_2_HPO_4_, NaHCO_3_, Na_2_CO_3_, and NaOH were purchased from Nacalai (Kyoto, Japan). Gliadin (from wheat), casein, ovalbumin, zein, sodium dodecyl sulfate (SDS), trifluoroacetic acid, 2,4,6-trinitrobenzene sulfonic acid (TNBSA), arginine, urea, and acetone were obtained from Sigma (St. Louis, MO, USA). Ethylenediamine was purchased from Thermo Fisher (Waltham, MA, USA). Polyethylene terephthalate (PET) films (4.5, 12, 23, 38, 50, and 75 µm thick) were obtained from Loyal Chemical Industrial (Kaohsiung, Taiwan). The amino-modified gliadin-binding aptamer (5′-NH_2_-CCAGTC TCCCGT TTACCG CGCCTA CACATG TCTGAA TGCC-3′, 40 nucleotides selected by Amaya-González et al. [15]) was obtained from PURIGO Biotechnology (Taipei, Taiwan). The RIDASCREEN^®^ Gliadin Competitive ELISA Assay (R7021) was supplied by R-Biopharm (Darmstadt, Germany). Commercial soy sauce samples were collected from local markets in Taipei, Taiwan. All chemicals were analytical grade and used directly without purification.

### 2.2. Capacitive Sensing Platform Construction

**Figure 1:**
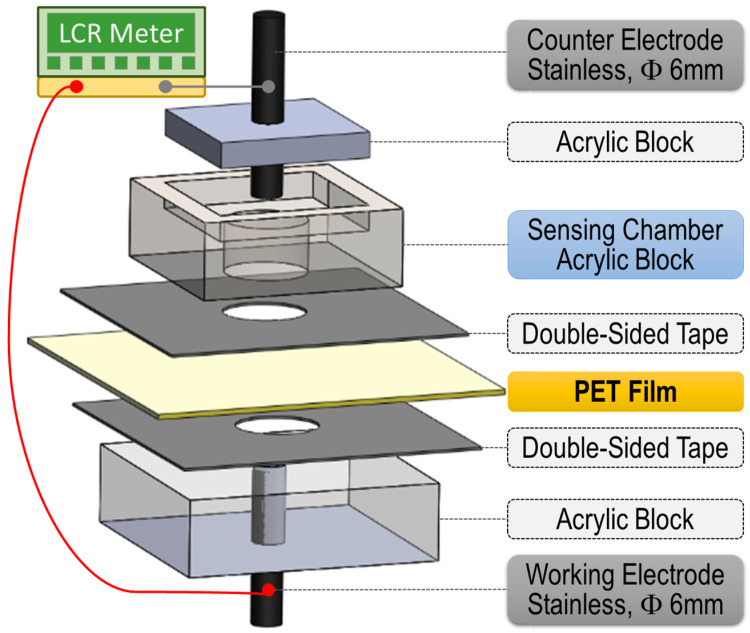
Exploded schematic of the custom-built capacitive sensing platform connected to an LCR meter.

As shown in Figure 1, the capacitive sensing platform consisted of two stainless-steel electrodes (6 mm diameter each). The counter electrode was centrally embedded in a top acrylic block (3 cm × 3 cm × 0.5 cm) and extended 0.5 cm downward. The working electrode was centrally embedded in a bottom acrylic block (5 cm × 5 cm × 1.5 cm). An intermediate acrylic block (4 cm × 4 cm × 1.5 cm) with a recessed cavity (3 cm × 3 cm × 0.7 cm) was placed between the two blocks to accommodate the top block and define a cylindrical sensing chamber (1 cm diameter, 0.8 cm depth; equivalent to drilling through the acrylic block). A PET film (6 cm × 6 cm, intentionally oversized for easy removal and re-fabrication) was sequentially cleaned in ethanol, acetone, and deionized water (3 min each) by ultrasonication. The cleaned PET film served as an insulating layer between the sensing chamber and the working electrode. The complete assembly was fabricated by stacking the intermediate block, PET film, and bottom block, which were secured using two layers of double-sided tape (3M™ 9671LE, St. Paul, MN, USA) featuring central 1 cm diameter holes. The electrodes were linked to an LCR meter (LCR-6300, GW Instek, Taipei, Taiwan). Capacitance was measured in a series-equivalent circuit model at 100 kHz with a 0.5 V bias.

### 2.3. Surface Amination of PET and Characterizations of Films

A total of 300 μL of 50% ethylenediamine was introduced into the sensing chamber, and the PET surface was aminated by heating at 55 °C for 15 min. The film was then rinsed with deionized water. The aminated region was subsequently excised, and its thickness was directly measured using a digital micrometer (Mitutoyo 293-100-10, 0.1 µm resolution, Kanagawa, Japan). Measurements were performed at multiple positions on each PET sample to ensure reproducibility, and the averaged values were used for analysis. Notably, the thickness values were obtained independently of capacitance measurements. The surface morphology and microstructure of the film samples were observed using a scanning electron microscope (SU8010, Hitachi, Tokyo, Japan) operated at 10 kV under high-vacuum conditions. Surface amino groups were quantified using the TNBSA method. The aminated PET film was dissolved in 0.2 mL of trifluoroacetic acid and diluted with 9.8 mL of carbonate buffer (0.1 M, pH 9.0), followed by pH adjustment to 9.0 using 1 M NaOH. An aliquot (0.5 mL) was then reacted with 0.25 mL of 0.01% TNBSA at 37 °C for 2.5 h. The reaction was terminated by adding 0.25 mL of 10% SDS and 0.125 mL of 1 M HCl. Absorbance was measured at 335 nm using a spectrophotometer (V-730, Jasco, Tokyo, Japan), and the amino group content was determined from a calibration curve prepared with ethylenediamine (diluted with 0.1 M carbonate buffer, pH 9.0) as the standard. Because ethylenediamine possesses two free amino groups that react with TNBSA, the quantified results were divided by two for normalization.

### 2.4. Aptamer Immobilization

An appropriate amount of lyophilized aptamer powder was weighed and dissolved in ultrapure water to obtain a stock solution at a concentration of 1 mM. The stock solution was stored at −4 °C to prevent degradation. For experiments, the aptamer stock solution was diluted to the desired concentration in PBS^+^ solution (0.1 M PBS, pH 7.0, with 1 mM MgCl_2_). The diluted aptamer solution was heated at 98 °C for 5 min, rapidly cooled to 4 °C for 5 min, and then incubated at 25 °C for 30 min to allow folding into its functional three-dimensional conformation. Next, 300 μL of 5% glutaraldehyde solution was added to the sensing chamber, where it reacted covalently with the surface NH_2_ groups on the PET film at ambient temperature for 2 h. The chamber was thoroughly rinsed with deionized water, followed by the addition of 100 μL of the prepared aptamer solution, which was allowed to react at 4 °C for at least 12 h to complete aptamer immobilization. Unbound aptamers were washed away, and 300 μL of 1 M glycine was added to block residual aldehyde groups on glutaraldehyde for 2 h, thereby minimizing nonspecific protein binding during subsequent real sample analysis.

### 2.5. Capacitive Aptasensing

A three-stage measurement protocol was implemented to verify the insulating integrity of the aptamer-functionalized PET film (stage 1) and enable reliable capacitive aptasensing (stages 2 and 3). In stage 1, baseline capacitance was recorded for 15 min in a background electrolyte of 270 μL PBS^+^ (0.1 M PBS, pH 7.0, with 1 mM MgCl_2_) and 30 μL of 70% ethanol (gliadin solvent). Capacitance measurement was then paused to rinse the sensing chamber with deionized water. After refilling the chamber with 270 μL PBS^+^ containing 30 μL of the gliadin sample (in 70% ethanol), measurement was restarted, and the system was incubated at ambient temperature for 30 min to allow specific aptamer–gliadin binding (stage 2). The chamber was then rinsed again (with measurement paused beforehand) to remove unbound and nonspecifically bound molecules. Finally, a 15 min capacitance measurement was performed in fresh background electrolytes (stage 3). The net decrease in capacitance (ΔC) between stage 1 and stage 3, using the mean of the final 5 min of continuous measurements from each, was calculated and used to construct the calibration curve. For the determination of the limit of detection (LOD), the lowest analyte concentration corresponding to an S/N ratio ≥ 3 was defined as the LOD. The S/N ratio was calculated by defining the signal as the net decrease in capacitance (ΔC) between stage 1 and stage 3, while the noise was taken as the mean of the final 5 min of continuous measurement in stage 3.

### 2.6. Analysis of Gliadin in Soy Sauce

Commercial soy sauce samples (1.0 mL) were mixed with 9.0 mL of 70% ethanol, ultrasonicated for 15 min, and then centrifuged at 20,000× *g* for 15 min. The resulting supernatant was analyzed by capacitive aptasensing and the gliadin ELISA assay. Supernatants from gluten-free soy sauces were spiked with gliadin standard to target concentrations and evaluated for recovery via capacitive aptasensing.

### 2.7. Aptasensor Regeneration

To evaluate the performance of the regeneration solution, a 100 μg/mL gliadin standard was subjected to the three-stage capacitive aptasensing protocol. After cleaning with deionized water, 300 μL of regeneration solution was introduced into the sensing chamber (maintained at 25 °C) for 1 min to dissociate the aptamer–gliadin complex. After subsequent cleaning, the aptasensing interface was re-exposed to 100 μg/mL gliadin using the same three-stage protocol. Regeneration efficiency was quantified as the percentage of residual activity, calculated by the following:

(1) $$\left({\Delta C}_{\mathrm{after}\;\mathrm{regeneration}}/{\Delta C}_{\mathrm{before}\;\mathrm{regeneration}}\right)\;\times \;100$$

## 3. Results and Discussion

### 3.1. Relationship Between PET Thickness and Capacitance Response Before Amination

**Figure 2:**
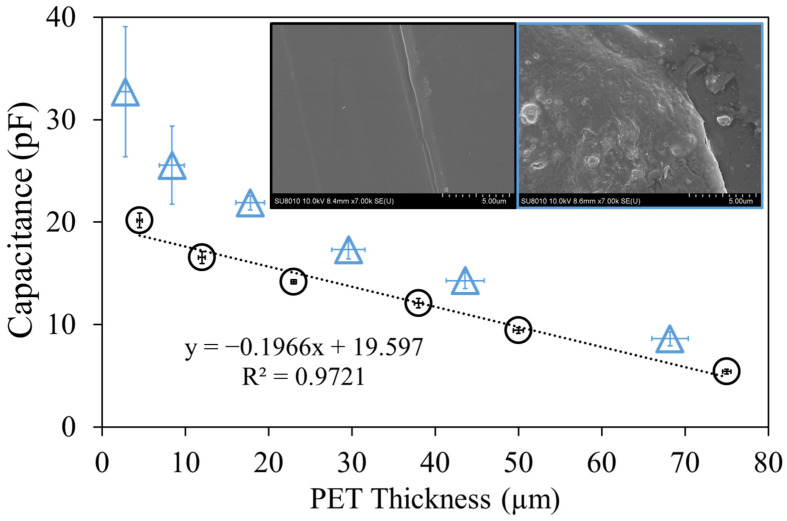
Relationship between PET thickness and capacitance response before amination (circles, N = 7) and after amination (triangles, N = 7). The thickness of each PET film was directly measured at five distinct, randomly selected locations using a digital micrometer, and the mean value was used for statistical analysis. Capacitance was monitored in background electrolytes for 15 min, using the mean of the final 5 min of continuous measurements for statistical analysis. The insets show SEM images of the PET films. The left image shows 23 µm PET before amination (a diagonal scratch was intentionally made with a blade to avoid a featureless uniform gray appearance and to clearly indicate it is a PET film), and the right image shows 23 µm PET after amination.

To confirm the capacitive response of the sensing platform aligns with theoretical predictions, we systematically investigated the effect of dielectric thickness on the measured capacitance (*C*). The parallel-plate capacitor equation is given by the following:

(2) $$C\;=\varepsilon_{0}\varepsilon_{r}A/d$$

where *ε*_0_ is the permittivity of the vacuum, *εᵣ* is the relative permittivity of the filling material, *A* is the overlapping electrode area, and *d* is the dielectric (PET film) thickness [25,26]. Thus, *C* varies inversely with *d*. As presented in Figure 2 (circle symbols), the measured capacitance decreases linearly with increasing PET film thickness over the range of 4.5–75 µm (R^2^ = 0.9721). These results confirm that PET films serve as a reliable and predictable insulating layer for subsequent biomolecular sensing applications.

### 3.2. Effect of Amination on PET Thickness and Capacitance Response

**Figure 3:**
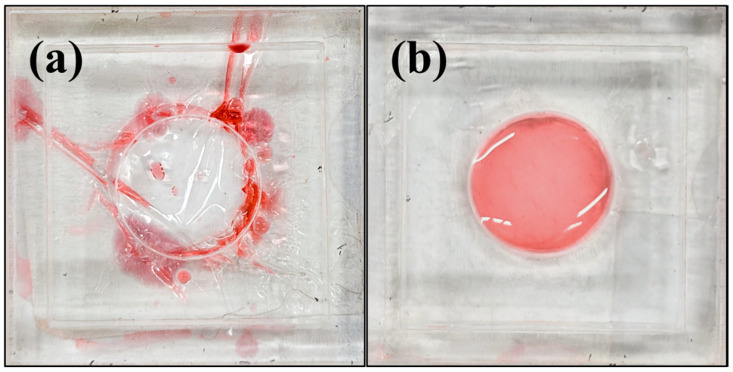
Top view of the sensing chamber with (**a**) a 12 μm thick and (**b**) a 23 μm thick PET film after 15 min of amination at 55 °C with 50% ethylenediamine. For easier observation, 300 μL of diluted red ink was introduced into the chamber.

Amination of PET films using ethylenediamine proceeds via a hydrolytic degradation mechanism, whereby ester bonds in the polymer backbone are cleaved, resulting in a progressive reduction in film thickness that scales with reaction duration [27,28,29,30]. As shown in the inset images of Figure 2, the PET surface transforms from a smooth and clean morphology (left) to a roughened surface (right), featuring irregular granular and flake-like structures that create a heterogeneous topography. Consequently, the film thickness becomes progressively thinner due to surface erosion and material removal. This effect is quantitatively illustrated by the triangular symbols in Figure 2. After 15 min of treatment at 55 °C in 50% ethylenediamine, the initial thicknesses of the 4.5, 12, 23, 38, 50, and 75 µm PET films decreased to 2.8, 8.4, 17.8, 29.6, 43.6, and 68.2 µm, respectively. As expected from the parallel-plate capacitor equation, the capacitance response increased as the film thickness decreased. However, prolonged amination leads to excessive degradation, which can compromise film integrity and cause solution leakage, as shown in Figure 3a. The 12 µm film exhibited pronounced structural deterioration, whereas the 23 µm film maintained its mechanical robustness (Figure 3b) throughout the degradative process due to its greater initial thickness. Such leakage disrupts the insulating barrier, allowing ionic ingress that alters the effective dielectric permittivity. This manifests as baseline instability in capacitance measurements, evidenced by the enlarged error bars for the 4.5 µm and 12 µm films after amination in Figure 2. These fluctuations undermine the reproducibility required for reliable aptasensing. Consequently, to minimize baseline drift and ensure consistent sensor performance during extended monitoring periods, the 23 µm PET film was selected for all subsequent experiments. Following a 15 min treatment at 55 °C in 50% ethylenediamine, the surface density of NH_2_ groups on the 23 µm aminated PET film was quantified by the TNBSA assay as 11.99 µmol/cm^2^ (N = 3). This relatively high density of NH_2_ functionalities provides abundant active sites for subsequent aptamer immobilization, thereby enhancing target accessibility. Consequently, it contributes to improved sensitivity and stronger signal transduction in gliadin detection.

### 3.3. Capacitive Aptasensing of Gliadin

**Figure 4:**
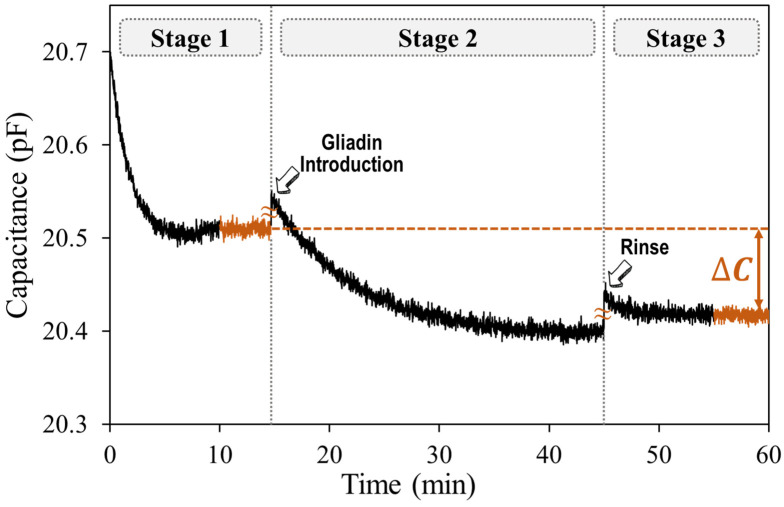
Typical capacitance response for the three-stage aptasensing of gliadin. Data were acquired following the procedures detailed in Section 2.5. Note that transitions between stage 1 and stage 2, and between stage 2 and stage 3, involve capacitance monitoring pauses for washing and restarting, resulting in non-continuous data traces. This characteristic capacitance response trend is consistently observed across successful capacitive aptasensing cases.

The typical response from the three-stage capacitive aptasensing of gliadin is shown in Figure 4. First, a 15 min baseline measurement checked the insulating integrity of the aptamer-functionalized PET film. Under well-insulated conditions, the final 5 min of capacitance monitoring at stage 1 showed excellent stability (standard deviation < 0.009 pF, N = 20). This low and stable background fluctuation contributed to improved signal reliability by minimizing baseline drift and noise, thereby supporting more consistent signal readout during subsequent aptasensing measurements. In contrast, poor PET film adhesion was mainly observed when the PET surface was insufficiently cleaned, when the bonding pressure was uneven, or when excessive amination led to interfacial cracking and subsequent solution leakage. These conditions could induce baseline shifts ranging from tens to thousands of pF, necessitating platform reassembly. This issue can be mitigated by performing a leakage check prior to measurement. At stage 2, gliadin was introduced into the sensing chamber, and the dynamic capacitive response was monitored for 30 min. Upon specific affinity binding between gliadin and the immobilized aptamer, the effective dielectric thickness increased. In addition, the displacement of high-dielectric-constant water molecules (εᵣ = 80) by lower-dielectric-constant gliadin molecules (εᵣ ≈ 20) decreased the relative permittivity of the sensing layer. According to the parallel-plate capacitor equation, these synergistic effects produced a continuous decline in capacitance until a steady-state plateau was reached at the end of stage 2 [23]. At stage 3, a rinsing step was performed to remove unbound gliadin molecules. The removal of nonspecifically bound molecules slightly reduced the effective dielectric thickness, leading to a modest increase in the steady-state capacitance value compared to that observed at the end of stage 2. This behavior is consistent with the theoretical prediction from the capacitor equation.

**Figure 5:**
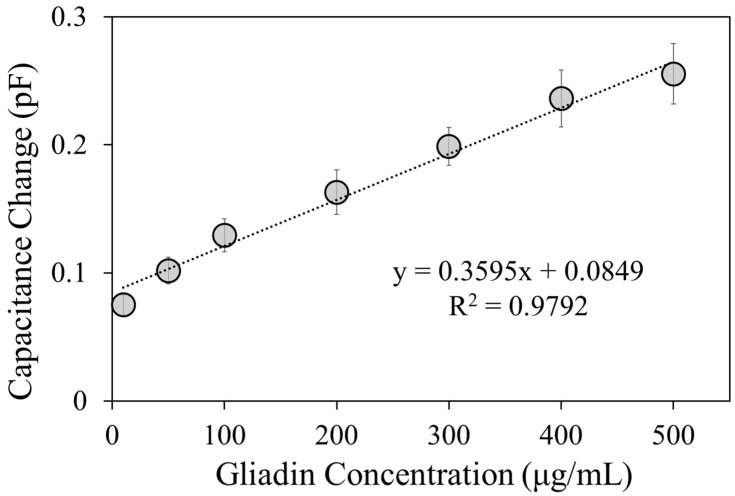
Calibration curve for capacitive aptasensing of gliadin using 10 μM immobilized aptamer. Error bars represent the standard deviation of seven replicates (N = 7).

To optimize the analytical performance of capacitive aptasensing for gliadin detection, we systematically evaluated immobilized aptamer dosages ranging from 1 to 20 μM. As summarized in Table 1, both sensitivity (slope) and linearity (range and R^2^) varied markedly with immobilization density. At lower dosages of 1 and 5 μM, the linear range was restricted to 10–300 μg/mL (R^2^ = 0.8642 and 0.9336, respectively), likely due to insufficient aptamer density that limited binding sites for higher gliadin concentrations. In contrast, 10 μM provided optimal performance, achieving the widest linearity (10–500 μg/mL), highest slope (0.3595), and best fit (R^2^ = 0.9792), reflecting an ideal balance of accessible binding sites for robust dielectric responses. Higher dosages (15 and 20 μM) maintained the 10–500 μg/mL range but showed diminished R^2^ values (0.9081 and 0.9066) and lower slopes (0.3132 and 0.3167), attributable to surface saturation, steric hindrance from aptamer overcrowding, and elevated nonspecific background signals. Thus, the 10 μM dosage was chosen for further experiments to ensure the highest sensitivity, linearity, and reproducibility, as demonstrated by the calibration curve in Figure 5, where the relative standard deviations were less than 10% at all tested concentration levels (N = 7). Under this optimal dosage of immobilized aptamer, the S/N ratio in response to 6.0 μg/mL gliadin was determined to be 4.28 ± 0.28 (N = 3), which corresponds to a limit of detection (LOD) for gliadin equivalent to 12 ppm of gluten content. This LOD value satisfies the European regulatory threshold of 20 ppm stipulated for verifying gluten-free food labeling, thereby demonstrating the sensor’s suitability for practical celiac disease risk assessment in food safety applications. Storage stability of the aptamer-immobilized sensing chamber was evaluated by incubation in PBS^+^ solution at 4 °C. Following a one-month storage period, the capacitive aptasensing response for 100 μg/mL gliadin exhibited only a negligible loss of less than 4.5% (N = 3), reinforcing the platform’s robustness for long-term use. Compared to Tsai et al. [23], the proposed capacitive aptasensor achieves comparable analytical performance. The core challenge of creating a functionalized interfacial layer was addressed through an inexpensive dielectric material and a straightforward surface modification method. This methodology greatly simplifies device fabrication and offers strong potential to advance capacitive sensing platforms.

### 3.4. Real Sample Determinations

**Figure 6:**
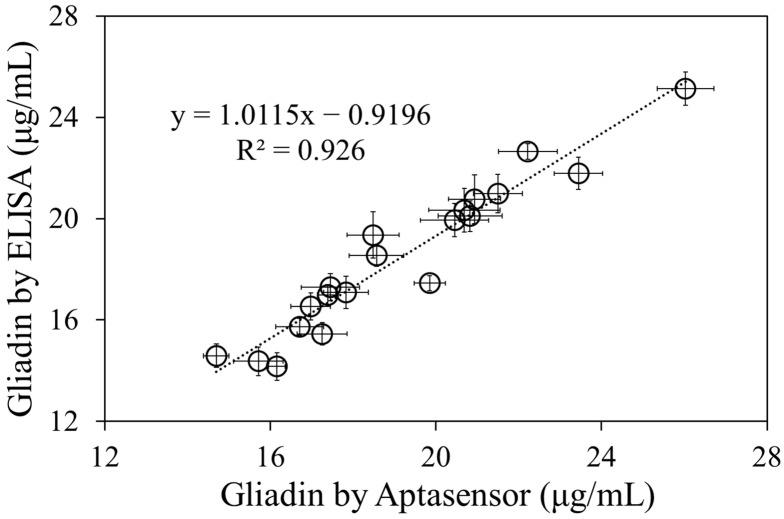
Correlation between aptasensor (N = 3) and commercial ELISA assay results (N = 3) for gluten-containing soy sauce samples.

Commercial gluten-containing soy sauce products exhibit diverse gluten levels due to variations in fermentation processes, in which wheat is frequently incorporated as the primary carbohydrate source, as well as differences in ingredient formulations [31]. To further validate the accuracy of our capacitive aptasensor in real samples, 20 such commercial gluten-containing soy sauce products were analyzed. The aptasensing results exhibited excellent correlation with a commercial ELISA assay (R^2^ = 0.926, N = 3, Figure 6), demonstrating its strong tolerance to complex soy sauce matrices with minimal interference. Furthermore, a spike recovery test was performed using supernatants from two commercial gluten-free soy sauces. Prior to analysis, their gluten-free status was confirmed by the ELISA assay, indicating that gluten levels in both samples were below the detection limit. The samples were then spiked with 30 or 100 μg/mL gliadin standard. The recoveries ranged from 91.7% to 105.7% (N = 3, Table 2), well within the acceptable limits for food safety assays. In addition, three common irrelevant food proteins, namely casein, ovalbumin, and zein, were tested to further investigate the selectivity of the aptasensor. Using the ΔC response induced by 100 μg/mL gliadin as the reference value of 100%, the corresponding ΔC signals generated by casein, ovalbumin, and zein at the same concentration were only 6.1%, 4.9%, and 7.6%, respectively (N = 3), indicating negligible cross-reactivity. These results collectively confirm the high selectivity and practical applicability of the developed aptasensor for real soy sauce analysis.

### 3.5. Reusability of Capacitive Aptasensor

**Figure 7:**
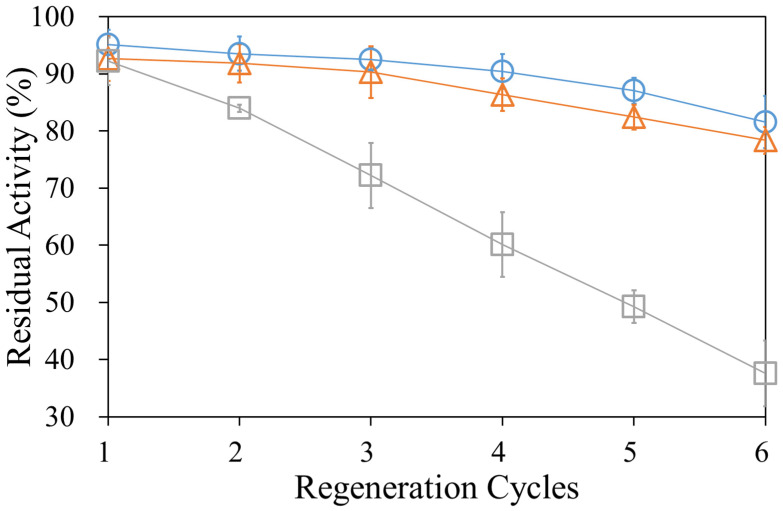
Multi-cycle regeneration performance of the aptasensor treated with 25 mM arginine at pH 9.0 (circles, N = 3), 25 mM arginine at pH 7.4 (triangles, N = 3), or 25 mM glycine at pH 2.4 (squares, N = 3) for 1 min at 25 °C.

Twelve solutions were evaluated for single-cycle regeneration efficiency by measuring the percentage of residual activity of the aptasensor (Table 3). Among them, 25 mM arginine at pH 9.0 yielded the highest residual activity of 95.1 ± 2.6%, closely followed by 25 mM arginine at pH 7.4 (92.7 ± 4.0%) and 25 mM glycine at pH 2.4 (92.2 ± 4.2%). These top performers disrupt gliadin–aptamer complexes through the complementary actions of arginine and glycine. Protein domains that bind nucleic acids are typically rich in positive charges, enabling electrostatic interactions with the aptamer’s negatively charged phosphate backbone. In addition, unpaired bases in loops and bulges facilitate specific recognition via numerous hydrogen bonds between nucleotide bases and amino acid side chains [32]. Arginine and glycine effectively compete for these sites and induce electrostatic repulsion, thereby dissociating the gliadin–aptamer complex without denaturing the aptamer. In contrast, high-salt solutions (2–5 M NaCl) and the denaturant urea yielded poorer results, primarily due to incomplete dissociation of bound gliadin or irreversible aptamer unfolding. For example, NaCl can promote reaggregation and crosslinking of gluten proteins [33], leading to multivalent gliadin aggregates that may fail to fully dissociate from the gliadin–aptamer complexes. In addition, exposure to high concentrations of urea can disrupt the higher-order structure of aptamers required for efficient target rebinding [34,35]. Multi-cycle testing further highlighted the superiority of arginine-based regeneration (Figure 7). Treatment with 25 mM arginine at pH 9.0 sustained an average residual activity of 81.6% after six cycles, while 25 mM arginine at pH 7.4 retained 78.4% after six cycles. Conversely, 25 mM glycine at pH 2.4 showed a sharper decline, dropping below 70% by cycle four, likely due to cumulative protonation-induced stress on the aptamer or electrode interface. Under alkaline conditions, arginine minimized degradation by better preserving aptamer conformation and electrode surface integrity. Overall, 25 mM arginine at pH 9.0 emerges as the optimal regeneration solution, enabling at least six reuses with >80% activity retention. This robustness, combined with a mild, short-duration treatment (1 min, 25 °C), enhances the sensor’s viability for on-site gliadin monitoring.

## 4. Conclusions

A novel label-free, reagentless capacitive aptasensor was developed for the sensitive and selective detection of gliadin, utilizing an aminated PET thin film as the functionalized insulating layer. Simple aminolysis with ethylenediamine enabled covalent immobilization of an amino-modified aptamer via glutaraldehyde crosslinking on the PET surface. An optimized initial film thickness of 23 μm ensured mechanical integrity, minimal baseline drift, and a stable capacitive response that aligned with the parallel-plate capacitor model. Under optimal conditions, the LOD met the European regulatory threshold of 20 ppm for gluten-free food labeling. Analysis of commercial soy sauces revealed strong agreement with ELISA results and satisfactory spike recoveries, confirming robustness in complex food matrices. These findings position aminated PET as a cost-effective, reusable dielectric interface for capacitive aptasensing. Beyond gliadin, this PET-based interface design offers a versatile platform for low-cost, reusable, and field-deployable capacitive biosensors targeting diverse analytes. The proposed approach provides a promising tool for on-site food safety monitoring, owing to its simplicity and reusability relative to conventional immunoassays. Future efforts will optimize the three-stage capacitive aptasensing process, potentially shortening inter-stage waiting times to enable detection within 30 min, along with device miniaturization and integration into portable formats for point-of-care applications.

## Figures and Tables

**Table 1:** Optimization of immobilized aptamer dosage for calibration curves in capacitive aptasensing of gliadin (N = 7).

Aptamer Dosage (μM)	Linearity (μg/mL)	Slope	R^2^
1	10–300	0.3155	0.8642
5	10–300	0.3282	0.9336
10	10–500	0.3595	0.9792
15	10–500	0.3132	0.9081
20	10–500	0.3167	0.9066

**Table 2:** Spike recoveries for gluten-free soy sauce samples by the developed aptasensor (N = 3).

Sample	Gliadin Spiked (μg/mL)	Gliadin Found (μg/mL)	Recovery (%)
Gluten-Free #1	30	31.7	105.7
	100	96.4	96.4
Gluten-Free #2	30	27.5	91.7
	100	93.9	93.9

**Table 3:** Percentage of residual activity of the aptasensor following a single regeneration cycle using various solutions for 1 min at 25 °C.

Regeneration Solution	Percentage Residual Activity (Average ± SD, N = 3)
2 M NaCl	42.2 ± 4.5
5 M NaCl	38.4 ± 5.4
6 M urea	74.3 ± 6.4
9 M urea	62.7 ± 5.4
25 mM arginine, pH 9.0	95.1 ± 2.6
25 mM arginine, pH 7.4	92.7 ± 4.0
25 mM arginine, pH 5.0	62.9 ± 6.2
25 mM arginine, pH 2.4	26.8 ± 4.3
25 mM glycine, pH 9.0	37.6 ± 6.1
25 mM glycine, pH 7.4	46.3 ± 6.4
25 mM glycine, pH 5.0	61.9 ± 5.6
25 mM glycine, pH 2.4	92.2 ± 4.2

## Data Availability

The original contributions presented in this study are included in the article. Further inquiries can be directed to the corresponding author.

## References

[1] Gujral N., Freeman H.J., Thomson A.B.R. (2012). Celiac disease: Prevalence, diagnosis, pathogenesis and treatment. World J. Gastroenterol..

[2] Singh P., Arora A., Strand T.A., Leffler D.A., Catassi C., Green P.H., Kelly C.P., Ahuja V., Makharia G.K. (2018). Global Prevalence of Celiac Disease: Systematic Review and Meta-analysis. Clin. Gastroenterol. Hepatol..

[3] Jain S., Lamba B.Y., Dubey S.K. (2024). Recent advancements in the sensors for food analysis to detect gluten: A mini-review [2019–2023]. Food Chem..

[4] Herrera-Quintana L., Navajas-Porras B., Vázquez-Lorente H., Hinojosa-Nogueira D., Corrales-Borrego F.J., Lopez-Garzon M., Plaza-Diaz J. (2025). Celiac Disease: Beyond Diet and Food Awareness. Foods.

[5] Wieser H., Segura V., Ruiz-Carnicer Á., Sousa C., Comino I. (2021). Food Safety and Cross-Contamination of Gluten-Free Products: A Narrative Review. Nutrients.

[6] Li Y., Liu Q., Godefroy S., Li J., Chen Y. (2025). Gluten Contamination of Labelled Gluten-Free Food Products Marketed in China. Foods.

[7] Atasoy G., Erdem I., Turhan M. (2026). Gluten contamination in manufactured gluten-free foods in Turkey: A five-year follow-up study. Food Addit. Contam. Part A Chem. Anal. Control Expo. Risk Assess..

[8] Studerus D., Lee A.R., Hugo T., Heim P., Jossen J., Scharl M., Zeitz J. (2026). Understanding cross-contamination in a gluten-free diet: A scoping review. Clin. Nutr. ESPEN.

[9] He Y., Yuan J., Khan I.M., Zhang L., Ma P., Wang Z. (2023). Research progress of aptasensor technology in the detection of foodborne pathogens. Food Control.

[10] Guo A., Zhang Y., Jiang M., Chen L., Jiang X., Zou X., Sun Z. (2025). Aptasensors for Rapid Detection of Hazards in Food: Latest Developments and Trends. Biosensors.

[11] Muhammed T.M., Ahmed A.T., Al-Hetty H.R.A.K., Rab S.O., Ballal S., Singh A., Devi A., Joshi K.K., Hadi A.M., Kaurshead R.S. (2025). Emerging aptasensor technologies for sensitive detection of food allergens: A comprehensive review. J. Food Compos. Anal..

[12] Roy J., Bag N., Roy S., Mondal D., Gong T., Mondal R., Guo B., Basu R., Das S. (2025). Aptasensing Technology and Its Potential Applications: Where Do We Stand?. Mol. Pharm..

[13] Shraim A.S., Majeed B.A.A., Al-Binni M.A., Hunaiti A. (2022). Therapeutic potential of aptamer–protein interactions. ACS Pharmacol. Transl. Sci..

[14] Ji C., Wei J., Zhang L., Hou X., Tan J., Yuan Q., Tan W. (2023). Aptamer–Protein Interactions: From Regulation to Biomolecular Detection. Chem. Rev..

[15] Amaya-González S., de-los-Santos-Álvarez N., Miranda-Ordieres A.J., Lobo-Castañón M.J. (2014). Aptamer Binding to Celiac Disease-Triggering Hydrophobic Proteins: A Sensitive Gluten Detection Approach. Anal. Chem..

[16] López-López L., Miranda-Castro R., de-los-Santos-Álvarez N., Miranda-Ordieres A.J., Lobo-Castañón M.J. (2017). Disposable electrochemical aptasensor for gluten determination in food. Sens. Actuators B Chem..

[17] Malvano F., Albanese D., Pilloton R., Matteo M.D. (2017). A new label-free impedimetric aptasensor for gluten detection. Food Control.

[18] Ramalingam S., Elsayed A., Singh A. (2020). An electrochemical microfluidic biochip for the detection of gliadin using MoS2/graphene/gold nanocomposite. Microchim. Acta.

[19] Svigelj R., Dossi N., Pizzolato S., Toniolo R., Miranda-Castro R., de-los-Santos-Álvarez N., Lobo-Castañón M.J. (2020). Truncated aptamers as selective receptors in a gluten sensor supporting direct measurement in a deep eutectic solvent. Biosens. Bioelectron..

[20] Svigelj R., Zuliani I., Grazioli C., Dossi N., Toniolo R. (2022). An Effective Label-Free Electrochemical Aptasensor Based on Gold Nanoparticles for Gluten Detection. Nanomaterials.

[21] Ham S.H., Kim E., Han H., Lee M.G., Choi Y.J., Hahn J. (2024). A label-free aptamer-based colorimetric biosensor for rapid gliadin detection in foods: A focus on pasta, bread and cookies. Anal. Methods.

[22] Qin Y., Zhang S., Qian J., Meng F., Yao J., Zhang M. (2024). Lable-free aptamer portable colorimetric smartphone for gliadin detection in food. Front. Bioeng. Biotechnol..

[23] Tsai C.-N., Lee C.-Y., Chen H.-Y., Hsieh B.-C. (2024). Parylene Double-Layer Coated Screen-Printed Carbon Electrode for Label-Free and Reagentless Capacitive Aptasensing of Gliadin. ACS Sens..

[24] Noel S., Liberelle B., Yogi A., Moreno M.J., Bureau M.N., Robitaille L., De Crescenzo G. (2013). A non-damaging chemical amination protocol for poly (ethylene terephthalate)—Application to the design of functionalized compliant vascular grafts. Mater. Chem. B.

[25] Inácio P.M.C., Guerra R., Stallinga P. (2025). A path toward transferable PEDOT: PSS-based capacitive sensors: Electrical modeling and fabrication. Sens. Actuators A Phys..

[26] Mathew S., Chintagumpala K. (2025). A review of recent progress in flexible capacitance pressure sensors: Materials design, printing methods, and applications. Adv. Compos. Hybrid. Mater..

[27] Bech L., Meylheuc T., Lepoittevin B., Roger P. (2007). Chemical Surface Modification of Poly (ethylene terephthalate) Fibers by Aminolysis and Grafting of Carbohydrates. J. Polym. Sci. A Polym. Chem..

[28] Hoang C.N., Dang Y.H. (2013). Aminolysis of poly (ethylene terephthalate) waste with ethylenediamine and characterization of α, ω-diamine products. Polym. Degrad. Stab..

[29] Lorusso E., Feng Y., Schneider J., Kamps L., Parasothy N., Mayer-Gall T., Gutmann J.S., Ali W. (2022). Investigation of aminolysis routes on PET fabrics using different amine-based materials. Nano Sel..

[30] Mohtaram F., Fojan P. (2025). From Waste to Value: Advances in Recycling Textile-Based PET Fabrics. Textiles.

[31] Diez-Simon C., Eichelsheim C., Mumm R., Hall R.D. (2020). Chemical and Sensory Characteristics of Soy Sauce: A Review. J. Agric. Food Chem..

[32] Choi S.-J., Ban C. (2016). Crystal structure of a DNA aptamer bound to PvLDH elucidates novel single-stranded DNA structural elements for folding and recognition. Sci. Rep..

[33] Ukai T., Matsumura Y., Urade R. (2008). Disaggregation and Reaggregation of Gluten Proteins by Sodium Chloride. J. Agric. Food Chem..

[34] Priyakumar U.D., Hyeon C., Thirumalai D., MacKerell A.D. (2009). Urea Destabilizes RNA by Forming Stacking Interactions and Multiple Hydrogen Bonds with Nucleic Acid Bases. J. Am. Chem. Soc..

[35] Aslanyan L., Ko J., Kim B.G., Vardanyan I., Dalyan Y.B., Chalikian T.V. (2017). Effect of Urea on G-Quadruplex Stability. J. Phys. Chem. B.

